# Abundance and diversity of nitrogen-removing microorganisms in the UASB-anammox reactor

**DOI:** 10.1371/journal.pone.0215615

**Published:** 2019-04-22

**Authors:** Rui Chen, Junqin Yao, Nuerla Ailijiang, Ruisang Liu, Lei Fang, Yinguang Chen

**Affiliations:** 1 College of Resources and Environmental Science, Xinjiang University, Urumqi, China; 2 College of Environmental Science and Engineering, Tongji University, Shanghai, China; Gifu University, JAPAN

## Abstract

Anaerobic ammonium oxidation is considered to be the most economical and low-energy biological nitrogen removal process. So far, anammox bacteria have not yet been purified from cultures. Some nitrogen-removing microorganisms cooperate to perform the anammox process. The objective of this research was to analyze the abundance and diversity of nitrogen-removing microorganisms in an anammox reactor started up with bulking sludge at room temperature. In this study, the ammonia-oxidizing archaea phylum Crenarchaeota was enriched from 9.2 to 53.0%. *Nitrosomonas*, *Nitrosococcus*, and *Nitrosospira*, which are ammonia-oxidizing bacteria, increased from 3.2, 1.7, and 0.1% to 12.8, 20.4, and 3.3%, respectively. *Ca*. *Brocadia*, *Ca*. *Kuenenia*, and *Ca*. *Scalindua*, which are anammox bacteria, were detected in the seeding sludge, accounting for 77.1, 11.5, and 10.6%. After cultivation, the dominant genus changed to *Ca*. *Kuenenia*, accounting for 82.0%. *Nitrospirae*, nitrite oxidation bacteria, decreased from 2.2 to 0.1%, while denitrifying genera decreased from 12.9 to 2.1%. The results of this study contribute to the understanding of nitrogen-removing microorganisms in an anammox reactor, thereby facilitating the improvement of such reactors. However, the physiological and metabolic functions of the ammonia-oxidizing archaea community in the anammox reactor need to be investigated in further studies.

## Introduction

The discovery of the anaerobic ammonium oxidation (anammox) process, a chemolithoautotrophic microbial process, took place in a denitrifying fluidized bed reactor in the early 1990s [1]. Under anaerobic conditions, the anammox reaction can directly convert ammonium to nitrogen gas using nitrite as an electron acceptor [2]. Stoichiometric ratios are considered to be an indicator of anammox processes [3]. According to previous studies, the corresponding molar ratios of the anammox process for NH_4_^+^ consumption, NO_2_^-^ consumption and NO_3_^-^ production are 1.00:1.32: 0.26, respectively [2]. Anammox is also an economical and effective method for nitrogen removal, since it was first discovered, compared to the traditional nitrification-denitrification method [1].

The anammox process is mediated by anammox bacteria and, according to data, six anammox bacterial genera, including *Ca*. *Brocadia* [4], *Ca*. *Kuenenia* [5], *Ca*. *Scalindua* [6], *Ca*. *Anammoxoglobus* [7], *Ca*. *Jettenia* [8], and *Ca*. *Anammoximicrobium* [9], have been enriched from samples collected from Wastewater Treatment Plants (WWTPs) and natural environments such as freshwater and marine areas [10]. All these genera belong to the same phylum, Planctomycetes [4]. The *Candidatus Scalindua* species are predominant in pristine freshwater ecosystems and marine environments, while the other five genera of anammox bacteria are mostly detected and enriched from the sludge of wastewater-impacted environments and WWTPs [11]. Anammox bacteria have not yet been purified from culture, indicating that they may coexist with other microorganisms [12]. Studies have found that anammox bacteria and ammonia-oxidizing bacteria (AOB) can coexist in a single reactor in which AOB oxidizes ammonium into nitrite, providing nitrite substance for anammox bacteria, while simultaneously consuming the dissolved oxygen (DO) and creating an anoxic environment for anammox bacteria [13]. Moreover, *Nitrosomonas*, which belongs to AOB, also exhibits anammox activity under anoxic conditions [14]. Studies have found that anammox bacteria and ammonia-oxidizing archaea (AOA) coexist in the Black Sea [15]. In low-oxygen environments, AOA can also provide nitrite to anammox bacteria [16].

Ammonia-oxidizing bacteria have commonly been reported in the anammox process, while the function of AOA has rarely been mentioned [17]. According to these reports, the growth of AOA can be encouraged by low DO values [18]. Ammonia-oxidizing archaea have an extremely high affinity towards ammonia, which makes them capable of achieving higher ammonia oxidation rates [19]. Therefore, AOA could be a better partner for anammox bacteria compared with AOB.

In this context, this research selected bulking sludge as seeding sludge to start up the anammox reaction at temperatures ranging from 20 to 31°C. The aim of this study was to investigate the abundance and diversity of AOA, AOB, anammox bacteria, nitrite oxidation bacteria (NOB), and denitrifying bacteria in the anammox reactor. The abundance and diversity of AOA in the UASB-anammox reactor started up with bulking sludge at room temperature were analysed for the first time.

## Materials and methods

### Anammox reactor

**Fig 1:**
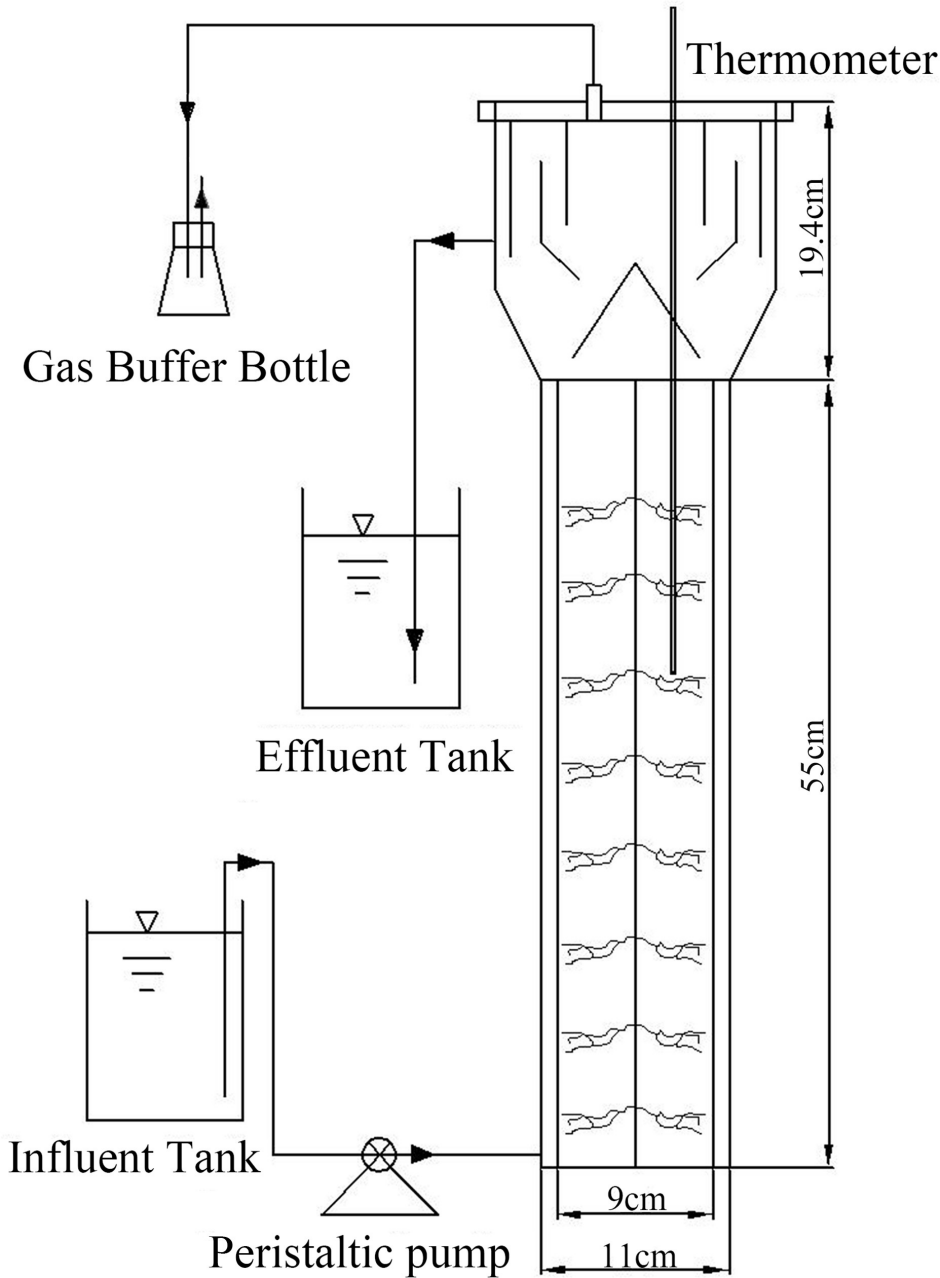
Schematic diagram of the UASB reactor.

The effective volume of the Up-flow Anaerobic Sludge Bed (UASB) reactor used in this study was 3.2 L (Fig 1). The reactor contained a membrane device and the surface was covered with an insulating layer to protect against light. Hydraulic retention time (HRT) was 16.9–74.6 h, and the reactor was operated at room temperature, which was maintained at 20–31°C.

### Seeding sludge and synthetic wastewater

The seeding sludge was obtained from the Changji WWTP (Xinjiang, China). The Sludge Volume Index (SVI) of the seeding sludge was 192 mL·g^-1^. At an SVI greater than 150 mL·g^-1^, the sludge is considered as bulking sludge [20].

Synthetic wastewater was used in this experiment and was composed of NH_4_Cl and NaNO_2_ as the main sources of ammonium and nitrite, without organic matter. The concentrations of NH_4_^+^-N and NO_2_^-^-N were 50 mg·L^-1^ and 70 mg·L^-1^. Other components included NaHCO_3_ (500 mg·L^-1^), MgSO_4_ (300 mg·L^-1^), CaCl_2_ (126 mg·L^-1^), KH_2_PO_4_ (30 mg·L^-1^); 1 mL of mother liquor of trace elements was added to each liter of synthetic water. The mother liquor of trace elements contained FeSO_4_ (5000 mg·L^-1^), MnCl_2_·H_2_O (990 mg·L^-1^), ZnSO_4_·7H_2_O (430 mg·L^-1^), CuSO_4_·H_2_O (250 mg·L^-1^), CoCl_2_·6H_2_O (240 mg·L^-1^), NiCl_2_·6H_2_O (190 mg·L^-1^), and H_3_BO_4_ (14 mg·L^-1^).

### Analysis

Two samples were collected from the UASB reactor. Sample A1 was collected from the seeding sludge on day 1, while sample A2 was collected on day 112 after successful anammox start-up. The samples for the microbial analysis were stored in the laboratory at -40°C and sent to Majorbio Bio-Pharm Technology Co. Ltd. (Shanghai, China) for DNA extraction, PCR amplification, and Illumina high-throughput sequencing.

Microbial DNA was extracted from two sludge samples that were collected from the UASB reactor using the FastDNA SPIN kit (Omega Bio-tek, Norcross, GA, U.S.) according to the manufacturer’s protocols. The final DNA concentration and purification were determined via a NanoDrop 2000 UV-vis spectrophotometer (Thermo Scientific, Wilmington, USA), whereas DNA quality was checked via 1% agarose gel electrophoresis. The target genes, primers, sequences, and PCR conditions are listed in Table 1. All PCR reactions were performed in triplicate in a 20 *μ*L mixture containing 4 *μ*L of 5x FastPfu Buffer, 2 *μ*L of 2.5 mM dNTPs, 0.8 *μ*L of each primer (5 *μ*M), 0.4 *μ*L of FastPfu Polymerase, and 10 ng of template DNA. The PCR products were extracted from a 2% agarose gel and further purified using the AxyPrep DNA Gel Extraction Kit (Axygen Biosciences, Union City, CA, USA). Subsequently, the products were quantified using QuantiFluor-ST (Promega, USA).

**Table 1:** Target genes, primers, and sequences used in the DNA amplification.

Target gene	Primer	Sequence (5’-3’)	PCR condition	References
AOA amoA	amoA-F	STAATGGTCTGGCTTAGACG	95°C for 3 min; 37 cycles of 30 s at 95°C, 30 s at 55°C, 45 s at 72°C; 10 min at 72°C	[21]
	amoA-R	GCGGCCATCCATCTGTATG		
AOB amoA	amoA-1F	GGGGTTTCTACTGGTGGT	95°C for 3 min; 35 cycles of 30 s at 95°C, 30 s at 55°C, 45 s at 72°C; 10 min at 72°C	[22]
	amoA-2R	CCCCTCGGGAAAGCCTTCTTC		
Anammox bacteria	Amx368F	TTCGCAATGCCCGAAAGG	94°C for 3 min; 32 cycles of 30 s at 94°C, 30 s at 52°C, 45 s at 72°C; 10 min at 72°C	[23]
	Amx820R	AAAACCCCTCTACTTAGTGCCC		
bacteria 16S rRNA	515F	GTGCCAGCMGCCGCGG	95°C for 3 min; 27 cycles of 30 s at 95°C, 30 s at 55°C, 45 s at 72°C; 10 min at 72°C	[24]
	907R	CCGTCAATTCMTTTRAGTTT		

Purified amplicons were pooled in equimolar and paired-end sequenced (2 × 300) on an Illumina MiSeq platform (Illumina, San Diego, USA) according to the standard protocols of Majorbio Bio-Pharm Technology Co. Ltd. (Shanghai, China). The raw reads were deposited in the NCBI Sequence Read Archive (SRA) database (Accession Numbers: SRP128971 and SRP167287).

### Data analysis

Data analysis was conducted using the i-Sanger platform (http://www.i-sanger.com/), provided by Majorbio Bio-Pharm Technology Co. Ltd. (Shanghai, China). The microbial phylotype richness levels were calculated using the Ace estimator and the Shannon diversity index. The Ace estimator, the Shannon diversity index, the Heip evenness index, and the coverage percentage were also calculated via the Mothur program version v.1.30.1. These analyses were performed using the R Programming Language software.

## Results and discussion

### Reactor performance

**Fig 2:**
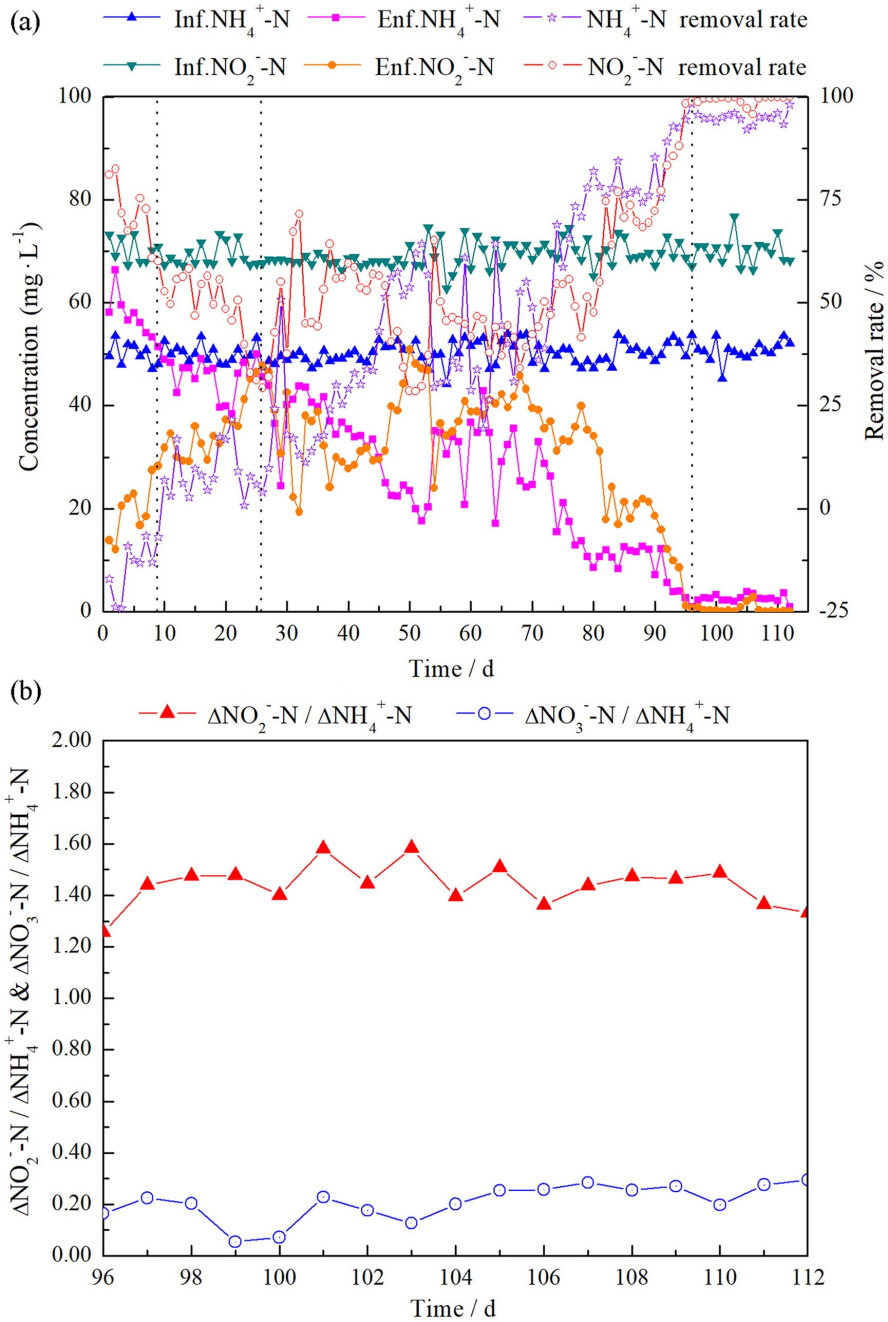
(a) Concentrations and removal efficiencies of NH_4_^+^-N and NO_2_^-^-N, (b) Stoichiometric ratios of the reactor during 96–112 d.

The concentrations of influent NH_4_^+^-N and NO_2_^-^-N were about 50.0 and 70.0 mg·L^-1^, respectively (Fig 2A). During days 1–9, the NH_4_^+^-N concentration of the effluent exceeded that of the influent, with the peak value of effluent NH_4_^+^-N in the reactor reaching 66.4 mg·L^-1^. This phenomenon was consistent with numerous previous studies and was named the “cell lysis phase” [25]. Denitrification was the dominant process, and the NO_2_^-^-N removal rate was 54.5–76.1%. The activity of anammox was not obvious.

During days 10–26, the NH_4_^+^-N removal rate was measured as 3.1–21.6%. Furthermore, the NO_2_^-^-N removal rate showed a downward trend and decreased from 60.1 to 29.3%, indicating that the activity of denitrifying bacteria had begun to decrease, as organic matter continued to be consumed [26]. Further, Ammonia and nitrite nitrogen were removed simultaneously with nitrate nitrogen production, indicating the occurrence of anammox activity. This stage was therefore termed the “anammox activity appearance period”.

During days 27–95, the removal rates of NH_4_^+^-N and NO_2_^-^-N gradually increased from 9.8 and 32.0% to 98.3 and 98.9%, respectively, while the NO_3_^-^-N production stabilized, with an average of 5.3 mg·L^-1^. This stage was named the “anammox activity elevation period”.

During days 96–112, the removal rates of NH_4_^+^-N and NO_2_^-^-N stabilized at 95.1 and 99.2%, respectively. The corresponding molar ratios of NH_4_^+^ consumption, NO_2_^-^ consumption, and NO_3_^-^ production were 1.00:1.41:0.21 (Fig 2B). The corresponding molar ratio of the last stage was close to the reported value (1:1.32:0.26) [2], indicating that this stage was the anammox activity stable period.

### Analysis of microbial community diversity

The total effective reads of the two sludge samples were 10,387–42,278 (Table 2). The coverage of each sample was more than 99%, indicating that the produced data was sufficient to cover all species. The Ace estimator represents the richness estimator, and larger values represent higher microbial community richness. The Shannon index is also a frequently used diversity index, and larger values represent higher microbial community diversity. The Heip index represents community evenness; higher values indicate a higher microbial community evenness. After cultivation, richness, diversity, and evenness of the AOA community were increased; AOA can actually be enriched under low-oxygen conditions. The diversity and evenness of the AOB community were also increased. In contrast, the diversity and evenness of the anammox bacteria were decreased, along with the richness, diversity, and evenness of the bacterial community.

**Table 2:** Summary of sequencing data for the two samples.

Microbial community	Sample	Reads	OUTs	Ace	Shannon	Heip	Coverage
AOA	A1	18,010	25	26	1.66	0.18	1.000
	A2	10,387	34	34	2.22	0.25	0.999
AOB	A1	19,141	34	35	1.18	0.07	0.999
	A2	33,073	31	35	1.86	0.18	0.999
Anammox bacteria	A1	28,573	9	9	1.49	0.43	1.000
	A2	40,557	5	8	0.48	0.15	0.999
Bacteria	A1	42,278	547	555	5.03	0.28	0.999
	A2	33,934	438	461	4.14	0.14	0.998

### Microbial community analysis

Based on previous studies, nitrogen-removing microorganisms are mainly AOA, AOB, NOB, denitrifying bacteria, and anammox bacteria (Table 3) [27–35]. Studies on AOA and anammox bacteria were mainly performed in natural environments such as freshwater lakes, rivers, or the Great Barrier Reef [27–30], while only a few studies on AOA were performed in lab-scale anammox reactors; AOB have mainly been studied in PN-A (partial nitritation/anammox) systems [31]. In contrast, anammox bacteria are relatively well studied [32–33]. According to previous studies, the phylum Planctomycetes contains all anammox bacterial genera [4], and NOB can compete with anammox bacteria for nitrite. In oxygen-poor environments, the metabolism of NOB is significantly suppressed [34]. The interaction between denitrifying bacteria and anammox bacteria has extensively been studied in lab-scale reactors [35], while studies on nitrogen-removing microorganisms in anammox reactors are rare (Table 3), especially in terms of richness and diversity.

**Table 3:** Studies of microbial communities involved in anammox.

Microbial community	Primer	Temperature (°C)	Research area	References
AOA	amoA-F/amoA-R	7–22	Limnetic sediments	[27]
		20	Pearl River	[28]
		27–35	South China Sea	[29]
		24–26	Sequencing batch reactor	[30]
AOB	amoA-F/amoA-R	-	PN-A systems	[31]
Anammox bacteria	Amx438F/Amx684R	13–33	UASB reactor	[32]
	Amx590F/Amx8715R	29–31	WWTPs	[33]
Bacteria	515F/907R	21–35	Lab-scale reactors	[34][35]

#### AOA community analysis at phylum and genus levels

Ammonium-oxidizing archaea can grow chemolithoautotrophically by aerobically oxidizing ammonia to nitrite [36], thereby contributing to ammoxidation. They are widely distributed in marine environments [37], soil [38], lakes [39], hot springs, and mining areas [40–41] and play an important role in the global nitrogen cycle. The AOA domain contains the phyla Euryarchaeota, Crenarchaeota, and Thaumarchaeota phyla [42], and AOA comprise an evolutionary group completely independent of AOB; however, AOB have traditionally been considered the main driver of ammonia oxidation [43], and researchers have overlooked the significant contributions of AOA to ammonia oxidation in a large range of environments [38]. It has been investigated that AOA are more active under certain extreme environmental habitats, such as low-oxygen environments [44].

**Fig 3:**
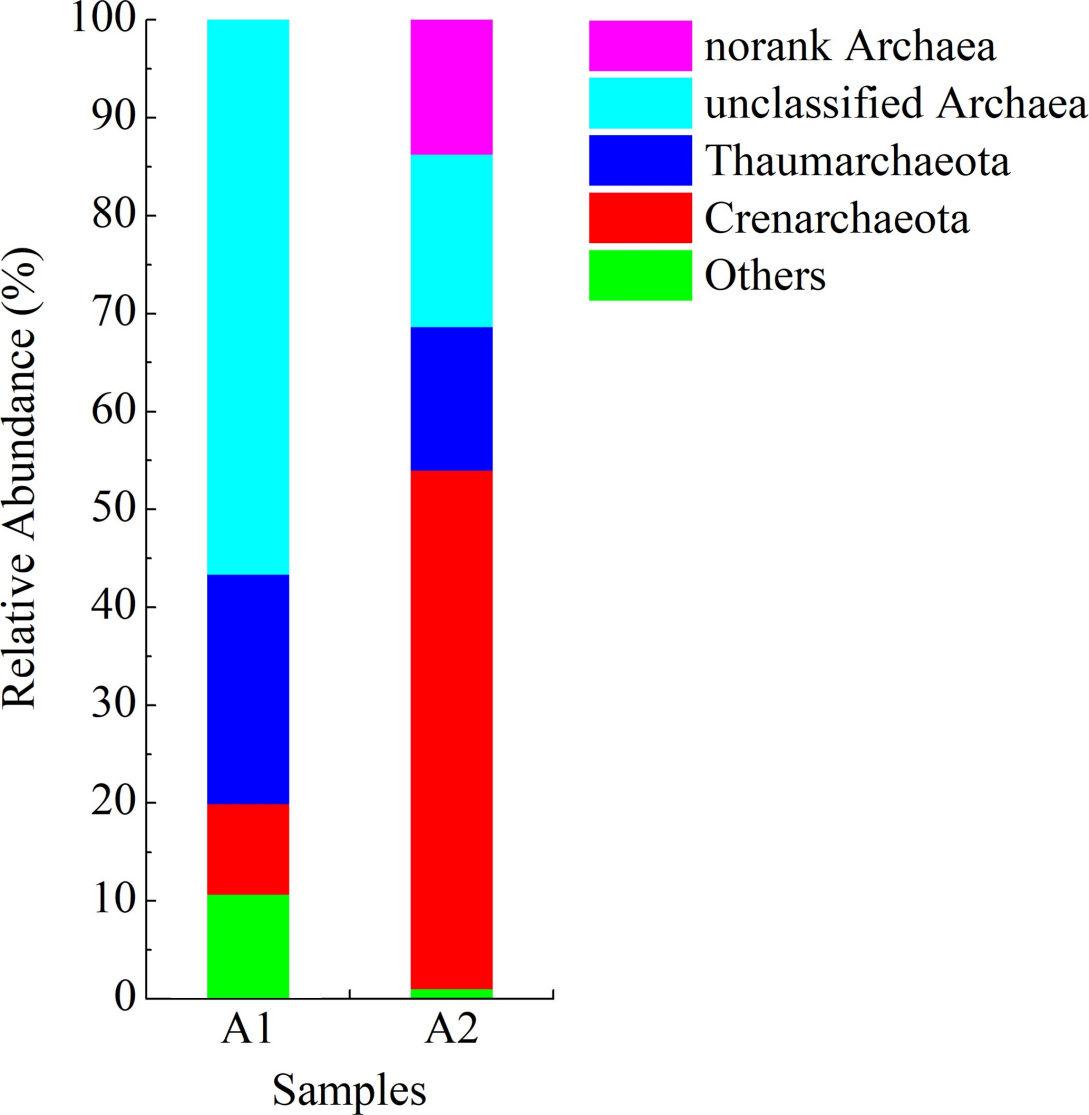
AOA community structure at the phylum level.

A total of 4 AOA phyla were detected from the two samples (Fig 3), including Crenarchaeota and Thaumarchaeota. The other two phyla were unclassified AOA phyla. The relative abundance of Crenarchaeota increased from 9.2% in the seeding sludge to 53.0% in the anammox sludge, while the relative abundance of Thaumarchaeota phylum decreased from 23.5 to 14.8%. The relative abundance of unclassified AOA genus *Crenarchaeota* increased from 9.2 to 53.0% (Table 4). The genera *Nitrosopumilus* and *Nitrososphaera* belong to the phylum Thaumarchaeota and significantly increased over time, accounting for 11.4 and 3.2% of the bacteria in the anammox sludge, respectively.

**Table 4:** Relative abundances of AOA genera in sludge samples.

**Microbial community**	**Phylum**	**Genus**	**A1 (%)**	**A2 (%)**
AOA	Crenarchaeota	*unclassified Crenarchaeota*	9.200	53.000
	Thaumarchaeota	*Nitrosopumilus*	0.700	11.400
		*Nitrososphaera*	0.000	3.200
		*unclassified Thaumarchaeota*	22.700	0.000

The phylum Crenarchaeota appeared to adapt well to the low-oxygen environment. A previous study has observed Thaumarchaeota and anammox bacteria in coexistence in different environments, particularly in anoxic water [45]. However, the relative abundance of the phylum Thaumarchaeota decreased in the reactor. The physiological and metabolic functions of the phylum Crenarchaeota need to be investigated in further studies.

#### AOB community analysis at phylum and genus levels

At the phylum level, the relative abundance of Proteobacteria increased from 26.4% in the seeding sludge to 90.7% in the anammox sludge, while the relative abundances of the genera *Nitrosomonas*, *Nitrosococcus*, *Nitrosospira*, and *Betaproteobacteria* increased from 3.2, 1.7, 0.1, and 0.3% to 12.8, 20.4, 3.3, and 38.2%, respectively (Table 5).

**Table 5:** Relative abundances of AOB genera in sludge samples.

**Microbial community**	**Phylum**	**Genus**	**A1 (%)**	**A2 (%)**
AOB	Proteobacteria	*Nitrosomonas*	3.171	12.847
		*Nitrosospira*	0.097	3.316
		*Nitrosococcus*	1.749	20.432
		*Betaproteobacteria*	0.268	38.215
		*unclassified Nitrosomonadaceae*	1.041	0.095
		*Nitrosomonadaceae*	20.006	15.802
		*unclassified Proteobacteria*	0.118	0.006

According to previous results, AOB can oxidize ammonia to nitrite, thereby providing nitrite for anammox and denitrification reactions [13]. In wastewater treatment processes, *Nitrosomonas* is frequently detected and shows anammox activity under anoxic conditions [14]. Previous studies have suggested that *Betaproteobacteria*, coexisting in anammox reactors, may consume organic compounds and trace amounts of O_2_, thus establishing suitable microenvironments for anammox bacteria [46].

#### Anammox bacterial community analysis at genus levels

**Fig 4:**
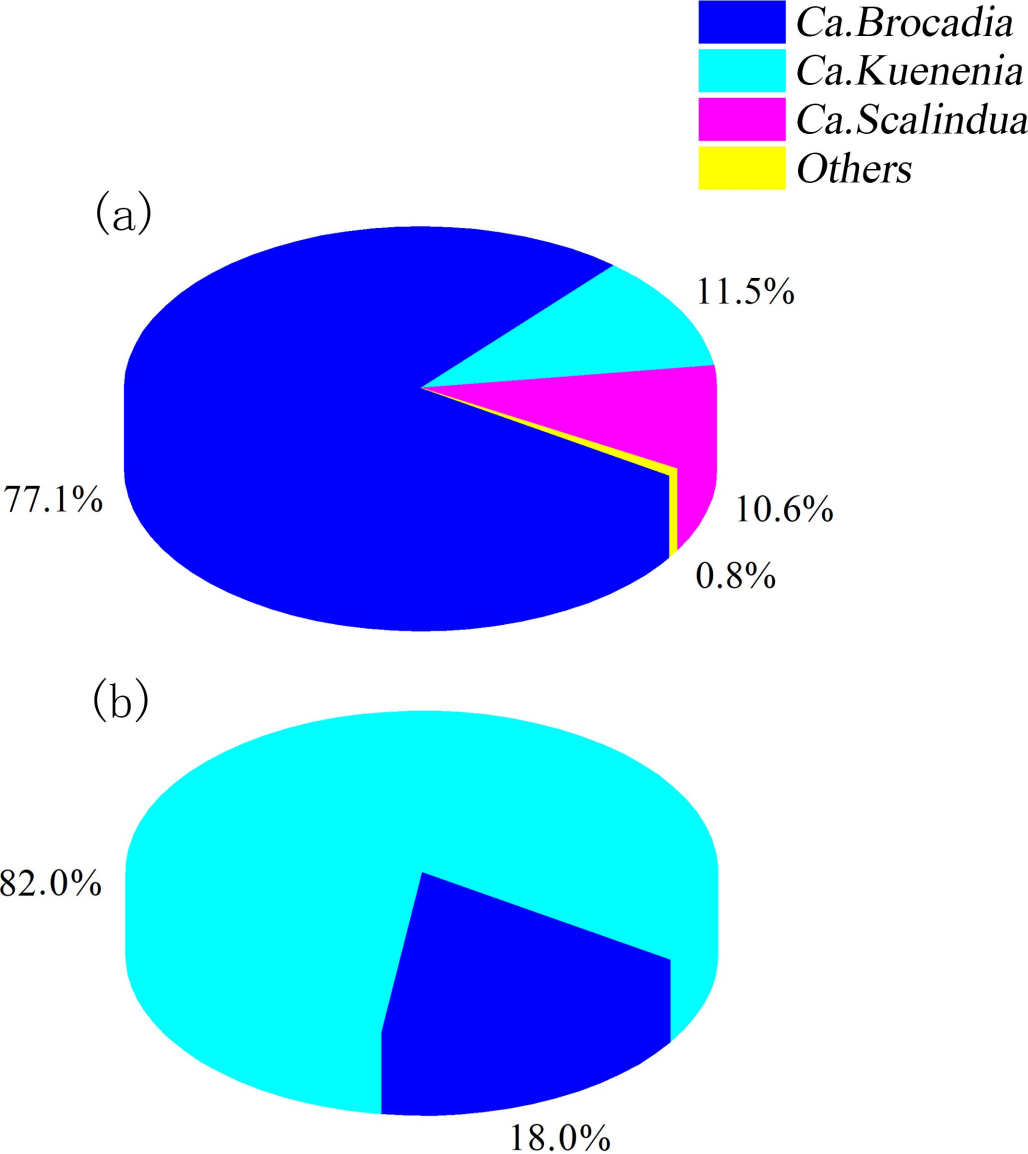
Anammox bacterial community structure at the genus level in A1 (a) and A2 (b).

The genera *Ca*. *Kuenenia*, *Ca*. *Brocadia*, and *Ca*. *Scalindua* were detected in the reactor (Fig 4), accounting for 11.5, 77.1, and 10.6% of the bacteria in the seeding sludge. After successful anammox start-up, *Ca*. *Kuenenia* became the dominant genus, accounting for 82.0%. The relative abundance of *Ca*. *Brocadia* decreased to 18.0%, while *Ca*. *Scalindua* disappeared. According to previous research, *Ca*. *Brocadia* and *Ca*. *Kuenenia* are common anammox bacterial genera in anammox reactors [47], and *Ca*. *Kuenenia* was the main species of anammox bacteria in a laboratory reactor fed with synthetic wastewater [48]. In another study, *Ca*. *Scalindua* is predominant in freshwater ecosystems and in marine environments [11].

#### Bacterial community analysis at phylum and genus levels

**Fig 5:**
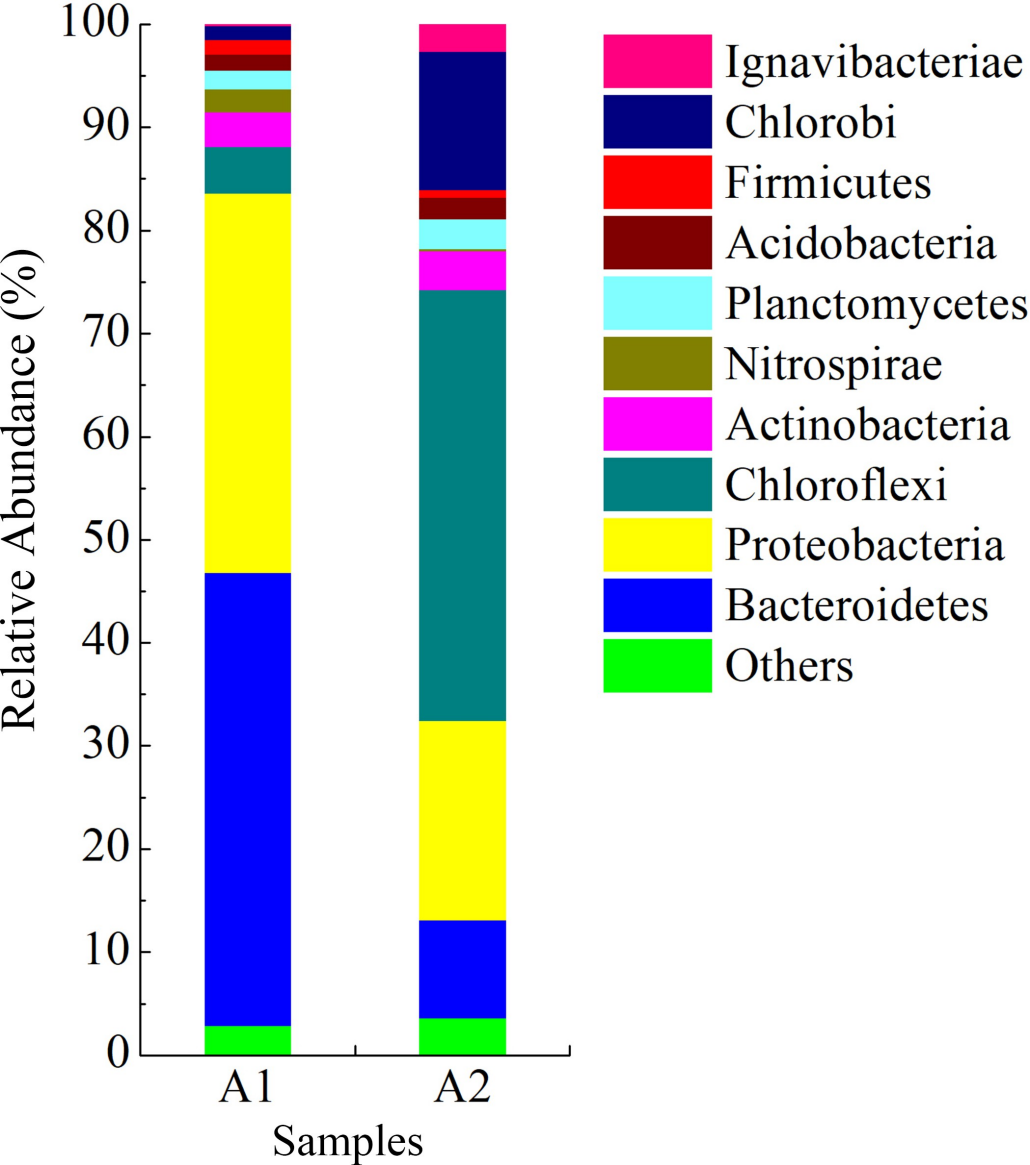
Bacterial community structure at the phylum level.

A total of 28 bacterial phyla were obtained in the two samples. The relative abundance of 10 bacterial phyla was greater than 1% in at least one sample (Fig 5). Bacteria were most abundant in the reactor. In the seeding sludge, the relative abundances of Bacteroidetes, Proteobacteria, Chloroflexi, Actinobacteria, Nitrospirae, Planctomycetes, Acidobacteria, Firmicutes, and Chlorobi were 43.5, 37.0, 4.6, 3.4, 2.2, 1.9, 1.5, 1.4, and 1.4%, respectively. In the anammox sludge, the relative abundances of Chloroflexi, Proteobacteria, Chlorobi, Bacteroidetes, Actinobacteria, Planctomycetes, Ignavibacteriae, Acidobacteria, Firmicutes, and Nitrospirae were 41.7, 19.0, 13.3, 9.6, 3.9, 3.0, 2.7, 2.2, 0.8, and 0.1%, respectively.

According to previous research, all anammox bacterial genera belong to the phylum Planctomycetes [4]. After the successful anammox start-up, the relative abundance of the phylum Planctomycetes increased from 1.9% in the seeding sludge to 3.0% in the anammox sludge. The phylum Chloroflexi is heterotrophic in microbial communities and prefers organic matter from dead anammox biomass as substrate [35]. Thus, anammox bacteria and the phylum Chloroflexi may interact within the anammox reactor [49]. The relative abundance of the phylum Chloroflexi increased from 4.6 to 41.7% overtime. Studies have reported that Chlorobi is an autotrophic bacterium and can thus be enriched through adaptation to the inorganic environment within the anammox reactor [50]. The relative abundance of the phylum Chlorobi increased from 1.4 to 13.3%. Nitrospirae was the only phylum of nitrite oxidation bacteria (NOB) in this study. The relative abundance of the phylum Nitrospirae decreased from 2.2 to 0.1%, which was consistent with the results of the anammox activity tests and confirmed the effective suppression of NOB in the anoxic environment [34].

**Fig 6:**
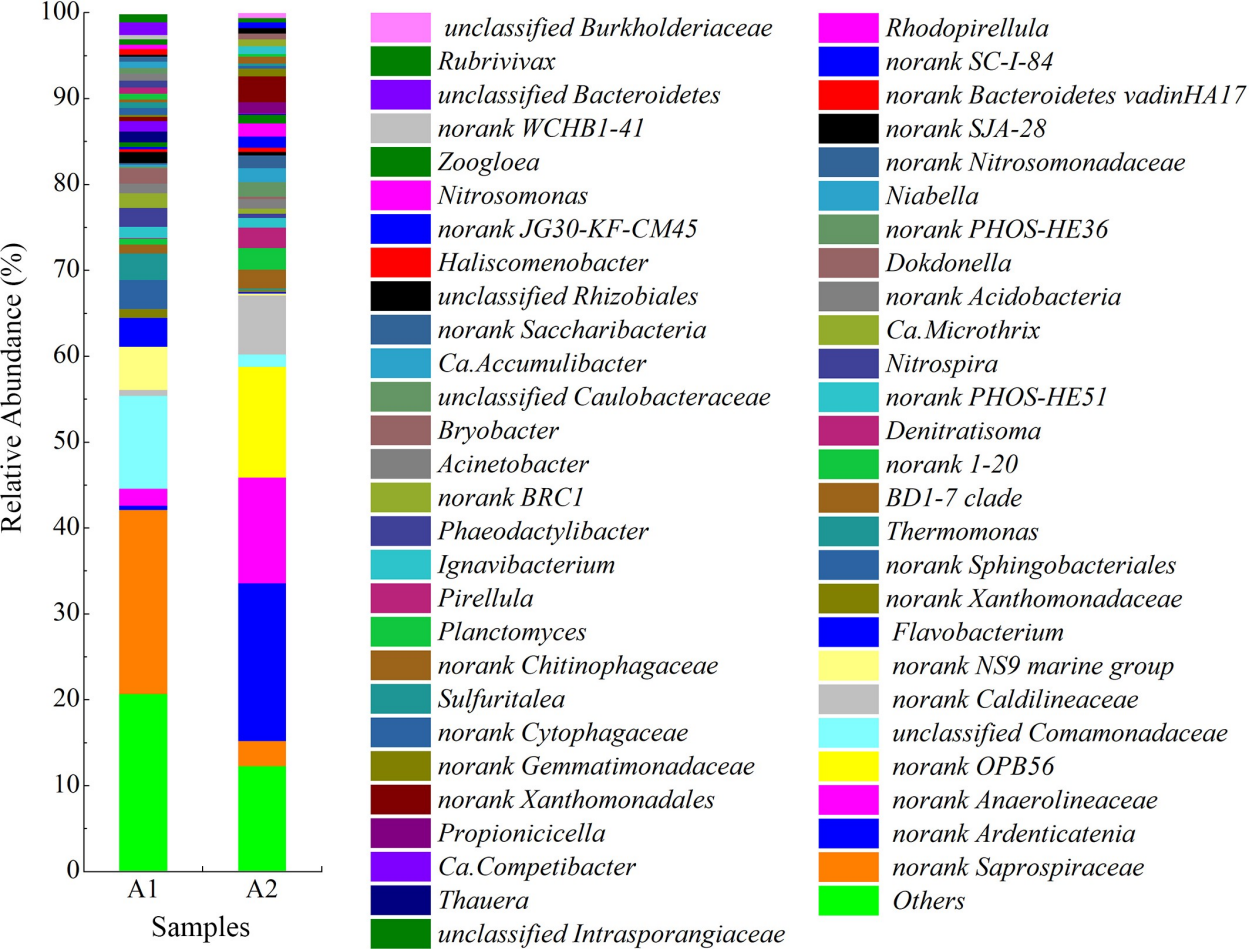
Bacterial community structure at the genus level.

A total of 294 bacterial genera were obtained in the two samples. The relative abundance of 54 bacterial genera was greater than 0.5% in at least one sample (Fig 6). The 54 bacterial genera accounted for 79.3–87.2% of the total bacterial effective sequences in each sample. The genera *Norank Ardenticatenia*, *norank Anaerolineaceae*, and *norank Caldilineaceae* belong to the bacterial phylum Chloroflexi, which increased from 0.5, 0.1, and 0.7% to 18.4, 12.9, and 6.9%. Research has shown that Saprospiraceae are aerobic bacteria and commonly cause bulking sludge [51]. After successful anammox start-up, the relative abundance of *norank Saprospiraceae* decreased from 21.4 to 2.9%, while that of the NOB genus *Nitrospira* decreased from 2.2 to 0.1%.

Research has shown that most denitrifying bacteria belong to the phyla Proteobacteria and Bacteroidetes [52–54]. The relative abundances of Proteobacteria and Bacteroidetes decreased from 37.0 and 43.5% to 19.0 and 9.6%, respectively. At the genus level, a total of 13 denitrifying genera of Proteobacteria and two denitrifying genera of Bacteroidetes were detected [53]. The relative abundance of denitrifying genera decreased from 12.9 to 2.1% (Table 6). According to a previous study, anammox activity inhibited the growth of denitrifying bacteria [55].

**Table 6:** Relative abundances of denitrifying bacterial genera in sludge samples.

**Microbial community**	**Phylum**	**Genus**	**A1 (%)**	**A2 (%)**
Denitrifying bacteria	Proteobacteria	*Thauera*	1.277	0.112
		*Dokdonella*	1.761	0.257
		*Dechloromonas*	0.446	0.012
		*Sulfuritalea*	0.747	0.349
		*Zoogloea*	0.575	0.012
		*Arenimonas*	0.061	0.325
		*Leptonema*	0.000	0.052
		*Thermomonas*	3.140	0.201
		*Comamonas*	0.480	0.008
		*Hydrogenophaga*	0.301	0.028
		*Pseudomonas*	0.343	0.020
		*Bdellovibrio*	0.046	0.000
		*Thiobacillus*	0.008	0.048
	Bacteroidetes	*Flavobacterium*	3.426	0.237
		*Terrimonas*	0.309	0.410
	Total	/	12.920	2.071

### Practical implications

Anammox bacteria have not yet been purified from cultures. Therefore, nitrogen-removing microorganisms cooperate to perform the anammox process. The abundances and diversities of AOA, AOB, anammox bacteria, NOB, and denitrifying bacteria greatly impact the nitrogen-removal efficiency of anammox systems. The results presented here offer new perspectives for the microbially mediated nitrogen removal in the practical application of anammox. This study demonstrated that the unclassified AOA genus *Crenarchaeota*, *Nitrosomonas*, *Nitrosococcus*, and *Nitrosospira* of AOB, and *Ca*. *Kuenenia* of the anammox bacteria are dominant nitrogen-removing microorganisms in this anammox reactor. In practical wastewater treatment systems, the role of AOA in the anammox process should be considered. In addition, the challenges in the current research and future work are to create suitable conditions for the balance among AOA, AOB, and anammox bacteria and the efficient inhibition of NOB.

## Conclusions

In this study, the diversity, richness, and evenness of AOA were significantly increased, while the unclassified AOA genus *Crenarchaeota* was enriched and increased from 9.2 to 53.0%, most likely because it adapted to the oxygen-poor environment. The AOB genera *Nitrosomonas*, *Nitrosococcus*, and *Nitrosospira* were enriched and increased from 3.2, 1.7, and 0.1% to 12.8, 20.4, and 3.3%, respectively. Three anammox bacterial genera, *Ca*. *Brocadia*, *Ca*. *Kuenenia*, and *Ca*. *Scalindua*, were detected. After cultivation, *Ca*. *Kuenenia* was enriched from 11.5 to 82.0% and became the dominant anammox bacterial genus, while *Ca*. *Brocadia* decreased from 77.1 to 18.0% and *Ca*. *Scalindua* disappeared completely. The NOB genus *Nitrospira* decreased from 2.2 to 0.1%, while denitrifying bacteria decreased from 12.9 to 2.1%.
